# The Effect of Rotigotine Extended-Release Microspheres Alone or With Celecoxib on the Inflammatory Pain

**DOI:** 10.3389/fphar.2020.594387

**Published:** 2020-10-30

**Authors:** Keke Li, Yijia Zhang, Enming Tian, Zikai Liu, Tian Wang, Fenghua Fu

**Affiliations:** ^1^ School of Pharmacy, Key Laboratory of Molecular Pharmacology and Drug Evaluation (Yantai University), Ministry of Education, Collaborative Innovation Center of Advanced Drug Delivery System and Biotech Drugs in Universities of Shandong, Yantai University, Yantai, China; ^2^ School of Pharmacy, Binzhou Medical University, Yantai, China

**Keywords:** analgesia, rotigotine extended-release microspheres, dopamine receptor, celecoxib, inflammatory pain

## Abstract

Clinical trials of rotigotine extended-release microspheres (RTGT-MS), which provides a sustained release of rotigotine for near 2 weeks *in vivo*, have been conducted in the treatment of Parkinson’s disease (PD). This study was to investigate the analgesic effect of RTGT-MS, and to know whether RTGT-MS have synergistic interaction with non-steroidal anti-inflammatory drug, celecoxib. The inflammatory pain model of rats was prepared by carrageenan-induced paw edema. The thermal and mechanical stimuli were applied and the hindpaw withdrawal latency (HWL) response was evaluated. Treatment with RTGT-MS increased the HWL in a dose-dependent manner. The ED50 of RTGT-MS was 24.68 ± 1.02 mg/kg. Isobolographic analysis shows that the combination of RTGT-MS and celecoxib resulted in a synergistic antinociceptive effect. Further results demonstrated that antinociceptive effect of RTGT-MS was accompanied with that PKA, cAMP, COX-2, and PGE2 levels were decreased. Chlorpromazine, a dopamine receptor blocker, not only weakened the analgesic effect of RTGT-MS, but also increased the levels of cAMP, PKA, COX-2, and PGE2. These findings provide a rationale for the combination of RTGT-MS and celecoxib in the treatment of PD, which may reduce the dose of celecoxib, thereby lowering the incidence of adverse effects and improving the pain management in PD patients.

## Introduction

Parkinson’s disease (PD) is a long-term, progressive neurodegenerative disease with typical clinical symptoms of static tremor, muscle rigidity, motor delay, and abnormal gait. Over six million patients with PD have been reported worldwide, and the prevalence of PD over the age of 65 is 1–2% (GBDPDS Collaborators, 2018). PD is now generally recognized as a complex disorder with clinical features encompassing motor, non-motor and neuro-psychiatric symptoms. Previous study shows that at least one non-motor symptom (NMS) has been observed in 99% of PD patients (Chaudhuri et al., 2006). It is well known that pain is a commonly reported non-motor symptom of PD, being prevalent in a large proportion of patients although the neurophysiology of pain in PD is not well understood. PD patients exhibited the incidence of pain ranges from 40% to 85% and the pain may significantly impair the quality of life of PD patients (Antonini et al., 2018; Broen et al., 2012).

PD patients may experience different types of pain, and Ford’s method classifies the pain into five types, including akathisia, radicular/neuropathic, musculoskeletal, central pain and dystonic pain, among which, musculoskeletal pain is the most common (Defazio et al., 2013). Musculoskeletal pain usually manifests as muscle pain, cramps, and articular/periarticular pain with an incidence of 10.6–69.8% (Ferini-Strambi et al., 2018; Lin et al., 2016). Otherwise the pain of PD can also be generally divided into two types from the perspective of pathophysiology: nociceptive pain and neuropathic pain. The pain caused by the actual or potential injury of non-neural tissue, as well as the pain caused by the activation of nociceptors, is called nociceptive pain. For example, dystonia related pains in toes, feet and less hands, is occurring during off-periods in early morning or biphasic beginning-of-dose or end-of-dose painful dyskinesias (Quinn et al., 1986; Schott, 1985) and the pain symptoms arising from skeletal or articulation deformation caused by Parkinson’s disease rigidity or abnormal posture (Ford, 2010). This pain is associated with rigidity, akinesia or dystonia in PD patients, which can lead to persisting stretching of muscles or joints, may result in inflammatory lesions, in turn, to result in the inflammatory pain.

Additionally, it should be point out that the pain in PD patients may be one associated with comorbid conditions, such as most commonly osteoarthritis and rheumatological diseases, which are generally recognized as the inflammatory pain.

Studies have shown that dopaminergic neuron losses may lead to nociceptive hypersensitivity. Levodopa and rotigotine have been used in the treatment of the pain symptoms in PD patients. Rotigotine, a dopamine receptor agonist, improves non-motor symptoms of PD patients (Storch et al., 2013), such as lowering the incidence of limb pain (Ghys et al., 2011). One study shows that rotigotine provides a clinical improvement in pain than placebo (Dworkin et al., 2009), and another study shows that, in patients with advanced PD, rotigotine transdermal patch improves chronic pain (Rascol et al., 2016). A good many of studies have shown that the cyclic adenosine monophosphate-protein kinase A (cAMP-PKA) signaling pathway plays an important role in the formation of pain and the continuous onset of pain (Malmberg et al., 1997).

Non-steroidal anti-inflammatory drugs (NSAIDs) are recommended to relieve pain in patients with PD who do not experience effective pain relief after treatment with the dopamine regimen (Perez-Lloret et al., 2012). Celecoxib can effectively attenuate acute pain and chronic pain by inhibiting peripheral and central cyclooxygenase-2 (COX-2). Although celecoxib may induce a great risk for elderly patients with PD by increasing the incidence of heart failure, stroke, myocardial infarction, and cardiovascular death (Solomon et al., 2008). It was still approved by the US Food and Drug Administration (FDA) for adult patients with osteoarthritis and rheumatoid (Krasselt and Baerwald, 2019).

Rotigotine extended-release microspheres (RTGT-MS, LY03003) provide a sustained release of rotigotine for near 2 weeks *in vivo*, and exhibit continuous dopaminergic stimulation for the treatment of PD with potential advantages for the treatment of mild and advanced PD patient in combination with l-DOPA (Wang et al., 2012). Clinical trials of RTGT-MS have been conducted in the treatment of PD, the pivotal clinical trials in USA, and Phase III clinical trials in China are ongoing. The purpose of this study was to investigate the analgesic effect of RTGT-MS, and to know whether RTGT-MS have synergistic interaction with non-steroidal anti-inflammatory drug, celecoxib, in the inflammatory pain model. If the combination of the two drugs produces synergistic analgesic effect, that may be clinically valuable because combination therapies may reduce the drug doses required for analgesic and thus reduce side effects.

## Materials and Methods

### Medicines and Reagents

RTGT-MS, as sterilized and lyophilized powder for injection, containing 22.58 mg of rotigotine per 100 mg, batch number 20170222, was provided by Luye Pharmaceutical Co., Ltd. (Luye Pharma), Yantai, China. The purity of rotigotine made by Luye Pharma and used to prepare RTGT-MS is 99.1%. RTGT-MS were prepared by an oil-in-water emulsion/solvent evaporation technique with poly (lactide-co-glycolide) (the mixture of PLGA 7525 4A and PLGA 5050 2A in the ratio of 7:3) as carrier materials. The molecular weights of PLGA 7525 4A (lactide/glycolide ratio, 75/25) and PLGA 5050 2A (lactide/glycolide ratio, 50/50) is 52,000 Da and 16,000 Da, respectively. RTGT-MS scanning electron microscopy analysis showed that the surface of the microspheres was smooth, spherical and nonporous. The mean particle size of the microspheres is about 70 μm. RTGT-MS exhibit a stable mean plasma level of 4.11 ± 1.59 ng/ml for near 2 weeks with a C_max_ of 6.27 ± 2.35 ng/ml on sixth day and a C_min_ of 1.39 ± 0.13 ng/ml at 14th day after intramuscular injection in rats (Wang et al., 2012). RTGT-MS was evaluated again *in vitro* before used. In the experiments, it was suspended in 2% carboxymethyl cellulose sodium and then injected intramuscularly into the rats in a volume of 1 ml/kg.

Chlorpromazine hydrochloride injection, containing 25 mg chlorpromazine hydrochloride per milliliter, was produced by Shandong Lukang Pharmaceutical Co., Ltd., Jining, China, batch number 170812. The purity of chlorpromazine hydrochloride used to prepare injection is 99.0%, according to Chinese Pharmacopoeia. Celecoxib capsules, containing 0.2 g celecoxib per capsule, was produced by Pfizer Pharmaceuticals LLC, NY, USA, batch number 20180327. The purity of celecoxib used to prepare capsules should meet United States Pharmacopeia standards. Carrageenan (Sigma-Aldrich, MO, USA, batch number: 20180413), COX-2 ELISA kit (Shanghai Enzyme-linked Biotechnology Co., Ltd., Shanghai, China, batch number: 20090428), PGE2 ELISA kit (Shanghai Enzyme-linked Biotechnology Co., Ltd., Shanghai, China, batch number: 20090423), PKA ELISA kit (Shanghai Enzyme-linked Biotechnology Co., Ltd., Shanghai, China, batch number: 20090430), cAMP ELISA kit (Shanghai Enzyme-linked Biotechnology Co., Ltd., Shanghai, China, batch number: 20090427), and sodium pentobarbital (Xiya Reagent, Linyi, China, batch number: F074) were purchased for experimental use. Celecoxib capsules content were suspended in 0.5% carboxymethyl cellulose sodium and were administered to rats via oral gavage in a volume of 10 ml/kg. Chlorpromazine hydrochloride (2.5 mg/kg) were diluted with saline and injected intraperitoneally. Carrageenan was dispersed in saline (2% m/v) and injected intraplantarly into the hindpaw of rats at a volume of 0.1 ml/paw. Sodium pentobarbital (50 mg/kg) were diluted with saline and injected intraperitoneally.

### Experimental Instruments

The experiments were performed using the following equipment, including Beckman Allegra X-22R Centrifuge purchased from Beckman Coulter, Inc., YLS-6B Intelligent Heat Panel Instrument from Yiyan Science & Technology Development Co., Ltd., Ugo Basile 7200 Plantar Test Apparatus from Ugo Basile, SONICS VCX130PB Ultrasonic Cell Disruptor from Sonics & Materials, Inc., and Rayto RT-6100 Enzyme Labeling Instrument from Rayto Life and Analytical Sciences Co., Ltd.

### Nociceptive Tests

To minimize the handling and measurement induced stress, the rats were acclimatized to the experimental conditions for 3 days. The responses of hindpaw withdrawal latency (HWL) to thermal and mechanical stimuli were evaluated according to the method as described elsewhere (Jin et al., 2010; Sun et al., 2003). The thermal stimulation was performed on the YLS-6B Intelligent Heat Panel Instrument, by placing the ventral surface of the rat hindpaw on a hot plate. The temperature of the plate was maintained at 52°C ± 0.2°C. The HWL to thermal stimulation were recorded as the time to hindpaw withdrawal. The HWL to mechanical stimulation was performed by the Randall Selitto Test on a Ugo Basile (Type 7200). The withdrawal response was measured after applying a wedge-shaped pusher to the dorsal surface of the hindpaw at a loading rate of 30 g/s. To avoid tissue damage, a cut-off limit of 15 s was applied (Zhang et al., 2017).

### Evaluation of the Analgesic Effect of Rotigotine Extended-Release Microspheres

Six-to-eight-week-old male Sprague-Dawley rats with a body weight of 180–220 g were purchased from the Experimental Animal Center of Luye Pharmaceutical Company, Yantai, China. All animals were kept under appropriate conditions at 24°C ± 1°C temperature, 60–70% relative humidity, and 12-h light/dark cycle. The rats were randomly divided into four groups, including the control group (control), the model group (model), the 40 mg/kg RTGT-MS group (RTGT-MS), and the 40 mg/kg RTGT-MS plus chlorpromazine hydrochloride group (RTGT-MS + chlorpromazine). Each group was composed of eight rats. In the control group and the model group, the rats received a single dose of 1 ml/kg vehicle (2% carboxymethyl cellulose sodium) via intramuscular infection. In the RTGT-MS group, the rats received a single dose of 40 mg/kg RTGT-MS via intramuscular injection, and the basal HWL were measured on the fourth day after the injection. In the RTGT-MS + chlorpromazine group, the rats were intraperitoneally injected with 2.5 mg/kg chlorpromazine twice a day for 4 days and received a single dose of 40 mg/kg RTGT-MS via intramuscular injection 30 min after the first chlorpromazine hydrochloride treatment. Then, the basal HWL were measured on the fourth day after the RTGT-MS injection. The inflammatory model of pain was established by the administration of 0.1 ml of 2% fresh suspension of carrageenan into both hindpaws of the rats via subplantar injection. In the RTGT-MS + chlorpromazine group, 2.5 mg/kg chlorpromazine hydrochloride was administered by intraperitoneal injection 30 min before the repeated measurement. The HWL of each animal were assessed at 3 h after inflammation induction.

### Determination of Cyclic Adenosine Monophosphate, Protein Kinase A, Cyclooxygenase-2, and Prostaglandin E2

After behavioral tests, the rats were anesthetized with 50 mg/kg sodium pentobarbital via intraperitoneal injection. The ankle joint of left inflamed hindpaw in rats were collected and then the rats were euthanatized. The paw was prepared in phosphate-buffered saline (PBS) with protease inhibitors at a concentration of 100 mg/ml after the skin was removed. To extract the tissue proteins, the samples were subject to centrifuge at 3,000 rpm for 10 min at 4°C, as described previously (Khan et al., 2013). The levels of cAMP, PKA, COX-2, and prostaglandin E2 (PGE2) in the inflamed tissues of left hindpaw from each animal were determined using an ELISA kit.

### Determination of ED50

As to the determination of ED50 of RTGT-MS, the rats (*n* = 8 in each group) received a single dose of RTGT-MS (3.9, 6.2, 10, 16, 25.4, and 40.7 mg/kg, respectively) via intramuscular injection. The rats receiving a dose of 1 ml/kg vehicle (2% carboxymethyl cellulose sodium) were used as the control. On the second day of the administration, the animals received a dose of 0.1 ml 2% fresh suspension of carrageenan at the site of the left hindpaw via subplantar injection. The HWL of each animal were assessed at 3 h after inflammation induction. Antinociception was quantified as the percentage of the maximum possible effect (%MPE), which was calculated using Eq. 1 (Afify and Andijani, 2017):

$$\text{\%MPE}=\frac{\left(\text{Postdrug}\;\text{latency}\right)-\left(\text{Predrug}\;\text{latency}\right)}{\left(\text{Maximum}\;\text{latency}\right)-\left(\text{Predrug}\;\text{latency}\right)}\times \frac{100}{1}$$ (1)

As to the determination of ED50 of celecoxib, the antinociceptive effect of celecoxib was firstly determined using regular rats. Celecoxib (12, 18, 27, 40.5, and 60.75 mg/kg) was administered intragastrically as a suspension in 0.5% carboxymethyl cellulose sodium solution (*n* = 4 in each group). The control rats received a dose of 10 ml/kg vehicle orally. The HWL of each animal were recorded at 0.5, 1, 1.5, 2, 3, 4, 5, 6, 7, 8, and 9 h after the administration of celecoxib or vehicle.

Subsequently, the dose-response curves of celecoxib were produced using the rats (*n* = 4 in each group) treated orally with different doses of celecoxib (12, 19.2, 30.72, 49.15, 78.64, and 125.83 mg/kg), and the ED50 was determined. In the control, the rats were treated with a dose of 10 ml/kg vehicle orally. At 30 min after administration, the left hindpaw of each animal was injected with 0.1 ml of 2% fresh carrageenan suspension. The HWL of each animal were assessed at 3 h after inflammation. Antinociception was also quantified as the %MPE, which was calculated according to Eq. 1.

As to the determination of the ED50 of co-administered RTGT-MS and celecoxib, ED50 fractions (1/2, 1/4, 1/8, and 1/16) of RTGT-MS and celecoxib were concurrently administered in a 1:1 ratio to establish the ED50 of the RTGT-MS-celecoxib combination (*n* = 6 in each group). For the control, the rats received a single dose of 1 ml/kg vehicle (2% carboxymethyl cellulose sodium) via intramuscular infection. The different doses of RTGT-MS were injected intramuscularly. On the fourth day, except for the control rats received a dose of 10 ml/kg vehicle (0.5% carboxymethyl cellulose sodium) orally, rats were treated orally with the different doses of celecoxib. At 30 min after celecoxib administration, the rats received 0.1 ml of 2% fresh carrageenan suspension into the left hindpaw via subplantar injection. At 3 h after inflammation, the HWL of each animal were assessed. The antinociceptive effects (%MPE) of the combination of RTGT-MS and celecoxib were determined according to Eq. 1.

### Isobolographic Analysis

The pharmacological effects of drug co-administration is often evaluated by isobolographic analysis (Fernández-Dueñas et al., 2012; Tallarida et al., 1989), by which, the dose of each drug is plotted on the *x* and *y* axes, respectively. The *isoboles* were generated by connecting the equi-effective doses of the combination. In addition, the additivity was also plotted by connecting the equi-effective doses of each individually administered drug. The mechanism of action of the co-administered drugs was evaluated by comparing the position of the isobole relative to the line of additivity (Ross et al., 2000). Statistically significant difference was then determined by juxtaposing the theoretical ED50 value with the experimental ED50 value (Zexp) (Tallarida et al., 1999). The synergism between the two drugs was determined according to the interaction index (*γ*), which was calculated by Eq. 2 (Tallarida, 2002):

$$\gamma =\frac{\text{a}}{\text{A}}+\frac{\text{b}}{\text{B}}$$ (2)

where A and B are the dose of RTGT-MS and celecoxib when administered alone, respectively, a and b are the dose of RTGT-MS and celecoxib in the combination, which produces the same antinociceptive effect. The interaction is additive, synergistic, and antagonistic when *γ* = 1, *γ* < 1, and *γ* > 1, respectively.

### Statistical Analysis

The data were presented as the mean ± standard error of the mean (SEM), which were then analyzed by one-way analysis of variance (ANOVA) and the least significant difference test for multiple comparisons (LSD). The ED50 (mean ± SEM) for RTGT-MS, celecoxib, and RTGT-MS plus celecoxib was calculated using GraphPad. A synergistic interaction between RTGT-MS and celecoxib was defined when the experimental ED50 was statistically significantly lower than the theoretical ED50. The differences between the experimental ED50 and the theoretical ED50 for drug combinations were analyzed using Student’s *t*-test. A *p* value of <0.05 was considered statistically significant.

## Results

### Analgesic Effects of Rotigotine Extended-Release Microspheres in the Carrageenan-Induced Inflammatory Pain Model

**FIGURE 1:**
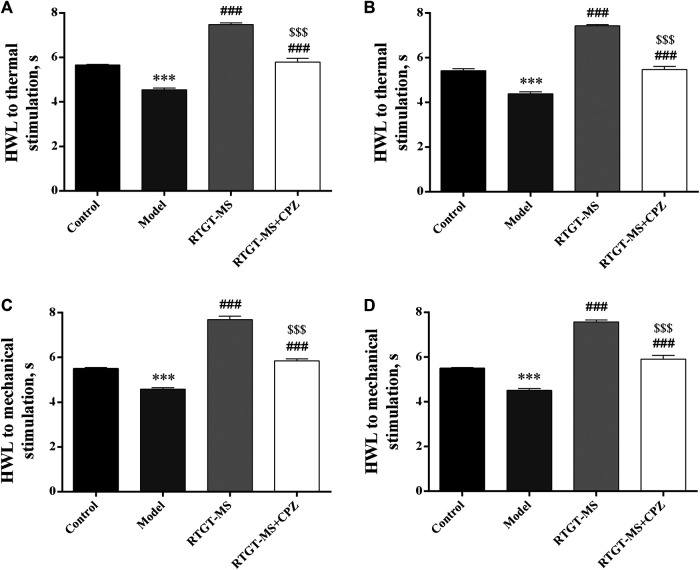
Effects of RTGT-MS and RTGT-MS plus chlorpromazine on the HWL in response to thermal **(A,B)** and mechanical stimulation **(C,D)** in rats with inflammatory pain. Left hindpaw HWL: **(A,C)**; right hindpaw HWL: **(B,D)**. The data are expressed as mean ± SEM (*n* = 8 in each group). HWL = hindpaw withdrawal latency. ***p < 0.001 vs. control group, ^###^ *p* < 0.001 vs. model group, ^$$$^ *p* < 0.001 vs. RTGT-MS 40 mg/kg group.

The analgesic effects of RTGT-MS on carrageenan-induced inflammatory pain are presented in Figure 1. The results show that the HWL of both hindpaws in response to thermal and mechanical stimulations were significantly reduced in the model group compared with those in the control group (*p* < 0.001). The HWL of both hindpaws in response to thermal and mechanical stimulations were significantly increased in the RTGT-MS group compared with those in the model group (*p* < 0.001). Compared with the RTGT-MS group, the HWL in RTGT-MS + chlorpromazine group were significantly decreased (*p* < 0.001).

### Effects of Rotigotine Extended-Release Microspheres on the Cyclooxygenase-2 and Prostaglandin E2 Levels

**FIGURE 2:**
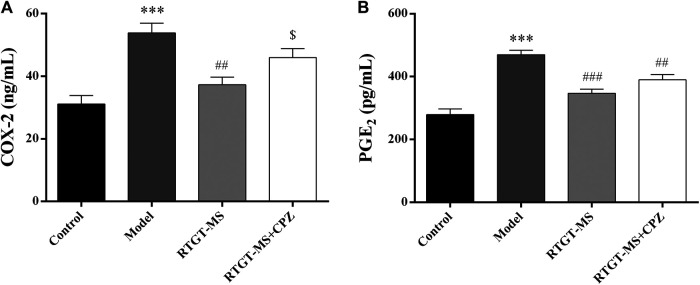
Effects of RTGT-MS and RTGT-MS plus chlorpromazine on the COX-2 **(A)** and PGE2 **(B)** level in the inflammatory pain model rats. The data are expressed as mean ± SEM of left hindpaw of rats (*n* = 6 in each group). ***p < 0.001 vs. control group, ^##^ *p* < 0.01, ^###^ *p* < 0.001 vs. model group, ^$^ *p* < 0.05 vs. RTGT-MS 40 mg/kg group.

It shows that the COX-2 and PGE2 levels were increased in the model group compared with those in the control group (*p* < 0.001). In the RTGT-MS group, the COX-2 and PGE2 levels were significantly decreased (*p* < 0.01 or *p* < 0.001). Compared with the RTGT-MS group, the COX-2 levels in the RTGT-MS + chlorpromazine group were significantly increased (*p* < 0.05) (Figure 2).

### Effects of Rotigotine Extended-Release Microspheres on Cyclic Adenosine Monophosphate and Protein Kinase A

**FIGURE 3:**
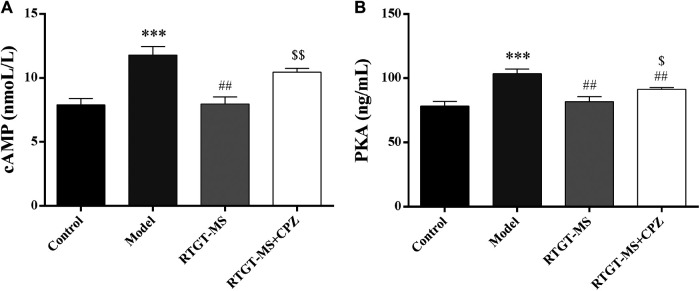
Effects of RTGT-MS and RTGT-MS plus chlorpromazine on the cAMP **(A)** and PKA **(B)** expression in inflammatory pain model rats. The data are expressed as mean ± SEM of left hindpaw of rats (*n* = 6 in each group). ***p < 0.01 vs. control group, ^##^ *p* < 0.01 vs. model group, ^$^ *p* < 0.05, ^$$^ *p* < 0.01 vs. RTGT-MS 40 mg/kg group.

The results show that the contents of cAMP and PKA were elevated in the model group compared with that in the control group (*p* < 0.001). The contents of cAMP and PKA were significantly decreased in the RTGT-MS group compared with that in the model group (*p* < 0.01). Compared with the RTGT-MS group, the contents of cAMP and PKA in the RTGT-MS + chlorpromazine group was significantly increased (*p* < 0.05 or *p* < 0.01) (Figure 3).

### ED50 of Rotigotine Extended-Release Microspheres in the Rat Model of Carrageenan-Induced Inflammatory Pain

**FIGURE 4:**
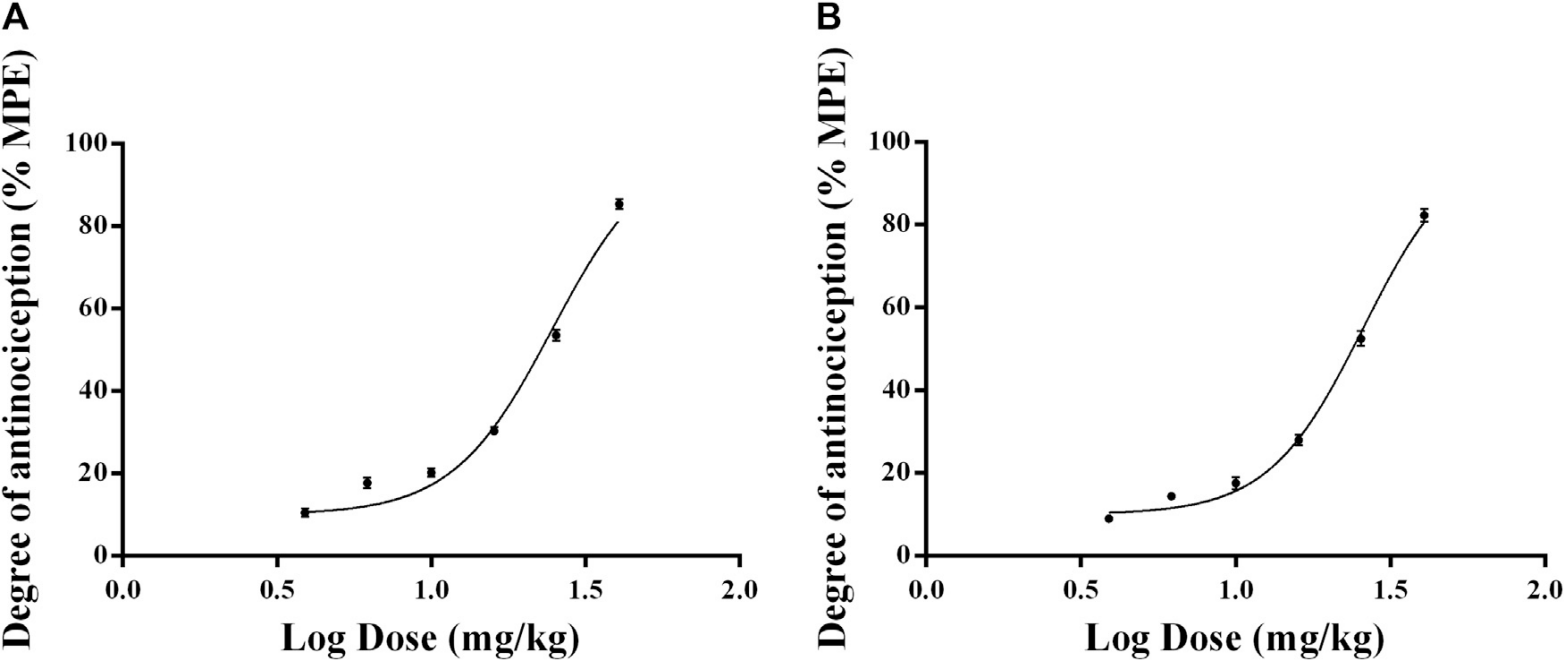
Dose-response curves of the antinociceptive effects of RTGT-MS in inflammatory pain model rats. The data are expressed as mean ± SEM of the maximum possible effect (%MPE) to thermal **(A)** and mechanical stimulation **(B)** of left hindpaw of rats (*n* = 8 in each group).

The results show that the withdrawal latency was increased in a dose-dependent manner after a single intramuscular injection of 3.9, 6.2, 10, 16, 25.4, and 40.7 mg/kg RTGT-MS. The ED50 ± SEM doses of intramuscular administered RTGT-MS were estimated to be 24.68 ± 1.02 mg/kg and 25.82 ± 1.02 mg/kg in response to thermal stimulation and mechanical stimulation, respectively (Figure 4).

### ED50 of Celecoxib in the Rat Model of Carrageenan-Induced Inflammatory Pain

**FIGURE 5:**
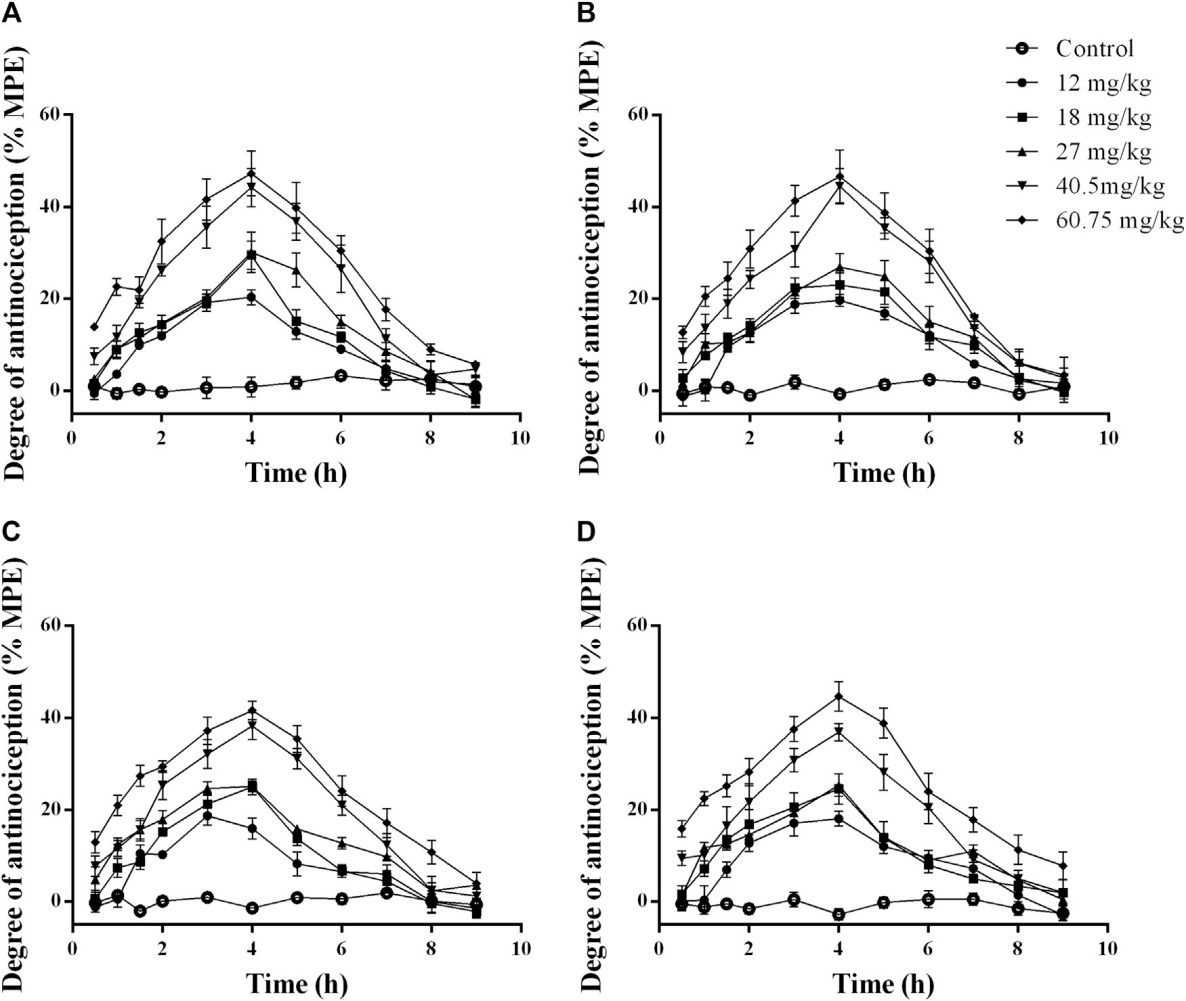
Time-response curves of the antinociceptive effects of celecoxib at different doses in normal rats. The data are expressed as mean ± SEM of the maximum possible effect (%MPE) to thermal **(A,B)** and mechanical stimulation **(C,D)** after intragastric administration of celecoxib in normal rats (*n* = 4 in each group). Left hindpaw HWL: **(A,C)**; right hindpaw HWL: **(B,D)**. HWL, hindpaw withdrawal latency.

**FIGURE 6:**
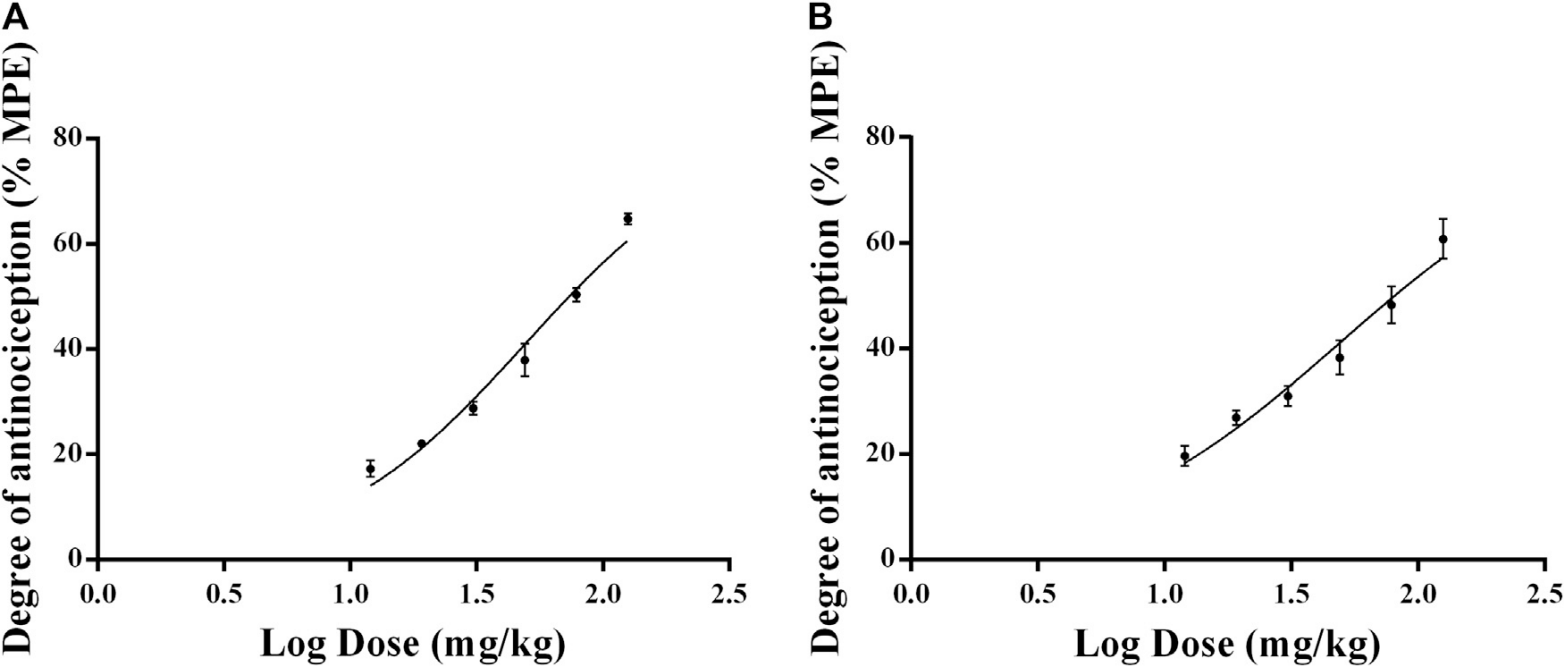
Dose-response curves of the antinociceptive effects of celecoxib in inflammatory pain model rats. The data are expressed as mean ± SEM of the maximum possible effect (%MPE) to thermal **(A)** and mechanical stimulation **(B)** of left hindpaw of rats (*n* = 4 in each group).

Figure 5 showed that the rats treated with 12, 18, 27, 40.5, and 60.75 mg/kg of celecoxib experienced significant antinociceptive effects. The analgesic effects were apparent from 0.5 h after celecoxib administration, with the strongest analgesic effect occurring at 3.5 h after administration and starting to decrease at 4 h. In the dose range of 12–60.75 mg/kg, the analgesic effect of celecoxib was dose-dependent. Therefore, when investigating the dose-effect relationship to determine the ED50 of celecoxib in the inflammatory pain model, the time point of 3.5 h after administration was appropriate. The ED50 doses for intragastric administration of celecoxib were calculated using the dose-response curves. Figure 6 showed the ED50 ± SEM were 49.31 ± 1.04 mg/kg for thermal stimulation and 48.97 ± 1.07 mg/kg for mechanical stimulation.

### ED50 of Co-Administered Rotigotine Extended-Release Microspheres and Celecoxib in the Rat Model of Carrageenan-Induced Inflammatory Pain

RTGT-MS (intramuscular injection) and celecoxib (gavage) were concurrently administered at a fixed ratio. The ED50 doses of RTGT-MS and celecoxib combinations were estimated from 12.70:24.63 mg/kg (1/2 of ED50), 6.35:12.32 mg/kg (1/4 of ED50), 3.18:6.16 mg/kg (1/8 of ED50), and 1.59:3.08 mg/kg (1/16 of ED50). The %MPE of the combination was analyzed (Tallarida and Raffa, 1996). The experimentally determined nociceptive ED50 ± SEM of RTGT-MS was 8.70 ± 1.04 mg/kg for thermal stimulation and 8.04 ± 1.04 mg/kg for mechanical stimulation. The experimentally determined ED50 ± SEM of celecoxib was 16.87 ± 1.03 mg/kg for thermal stimulation and 15.59 ± 1.04 mg/kg for mechanical stimulation. The theoretical additive ED50 value with 95% confidence intervals (95% CI) of RTGT-MS was 12.77 (10.41–15.13) mg/kg for thermal stimulation and 13.10 (10.84–15.36) mg/kg for mechanical stimulation. The theoretical additive ED50 value with 95% CI of celecoxib was 24.80 (22.44–27.16) mg/kg for thermal stimulation and 25.44 (23.18–27.70) mg/kg for mechanical stimulation.

**FIGURE 7:**
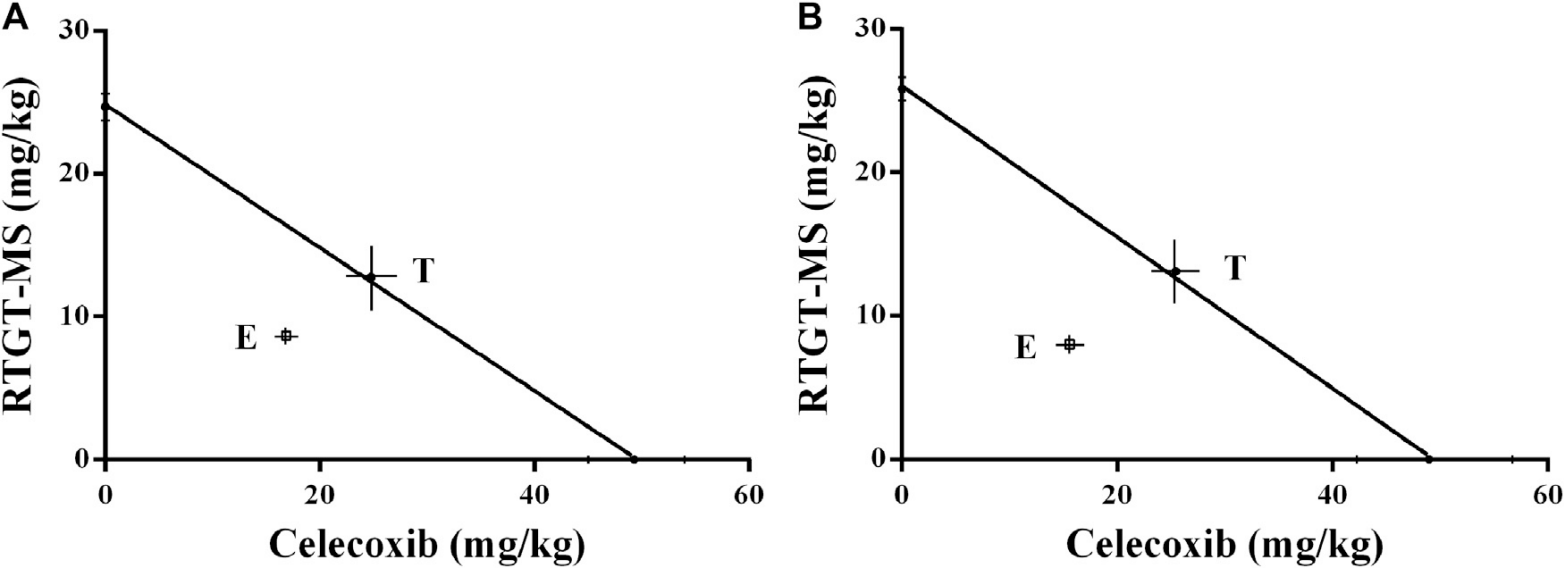
Isobologram antinociceptive synergistic interaction between RTGT-MS-celecoxib co-administration in inflammatory pain model rats. **(A)** thermal stimulation of left hindpaw of rats; **(B)** mechanical stimulation of left hindpaw of rats. The point E is the observed ED50 of the co-administered drugs, and the theoretical additive point (T) in the middle of the theoretical additive line was obtained from ED50 for each drug.

By isobolographic analysis, our results show that there was a synergistic effect between RTGT-MS and celecoxib in the model of inflammatory pain (Figure 7). Because the doses of RTGT-MS and celecoxib necessary to produce a 50% MPE were significantly less than those calculated to be necessary for an additive interaction. The interaction determined by thermal stimulation was consistent with that by the mechanical stimulation. The point E is the observed ED50 of the co-administered drugs, and the theoretical additive point (T) in the middle of the theoretical additive line was obtained from ED50 for each drug (Figure 7). The experimental ED50 is significantly different from the theoretical ED50 and located below the additive line, indicating there is a significant synergistic effect (*p* < 0.05). Our results also show that the potency of the analgesic was increased when the drugs were co-administered (*γ* = 0.6945 for thermal stimulation and *γ* = 0.6296 for mechanical stimulation).

## Discussion

Parkinson’s disease is a common chronic neurodegenerative disease with severe clinical manifestations including motor symptoms and non-motor symptoms. Pain is one of the major non-motor symptoms of PD (Defazio et al., 2013; Ford, 2009; Ford et al., 1996; Wen et al., 2012), and it may also be associated with comorbid rheumatological diseases, osteoarthritis, etc. No matter in PD itself or comorbid conditions some pain in PD patients is associated with inflammatory one. It has been reported that PD-related nociceptive pain can possibly be effectively treated by dopaminergic agents, such as levodopa or dopamine agonists or by anti-inflammatory analgesic, such as NSAIDs (Perez-Lloret et al., 2012).

As to the inflammatory pain, carrageenan induced inflammatory edema in rat has often been used to investigated the efficacy and mechanism of analgesic agents. In this study, the HWL in response to thermal and mechanical stimulation were determined to evaluate the analgesic effect of rotigotine, and its synergistic interaction with NSAIDs, celecoxib. Extended-release of dopamine agonists has been a promising method for the treatment of PD by offering many clinical advantages, such as, constant plasma drug concentrations and continuous dopaminergic receptor stimulation.

In the experiments, RTGT-MS group, the HWL of both hindpaws in response to thermal and mechanical stimulation were significantly increased compared with those of the model group (*p* < 0.001). The RTGT-MS exerted significant antinociceptive effects in this rat model of inflammatory pain. It is most possible that RTGT-MS could have continuous analgesic effects although its effect was observed at 4 days after RTGT-MS administered. RTGT-MS exhibited sustained release of rotigotine and efficacy for near 2 weeks in rats. The cardioprotective effects of RTGT-MS may maintained for 14 days after a single RTGT-MS administration (Lv et al., 2019; Wang et al., 2012).

Chlorpromazine is an antagonist for D1, D2, D3, and D5 receptors (Tadori et al., 2011). The antinociceptive effects of RTGT-MS in the inflammatory pain models were partially eliminated after chlorpromazine administration, which suggests that the antinociceptive effects of RTGT-MS may be mediated by these receptors at least partly.

It is generally recognized that one of the pathways that is regulated by dopamine receptors is the AC-cAMP-PKA pathway (Beaulieu et al., 2015). In recent years, it has been found that the cAMP-PKA signaling pathway is widely involved in the formation of hyperalgesia and the regulation of nociceptive information. The cAMP-PKA signaling pathway not only mediates peripheral pain sensitization induced by inflammatory mediators (Lewin and Walters, 1999; Malmberg et al., 1997), but also regulates neuropathic pain (Huang et al., 2012; Song et al., 2006; Zheng et al., 2007). As a D1/D2/D3 receptor agonist, rotigotine improves motor function in PD patients (Chen et al., 2017), and one study has shown that by coupling with the D2 receptor and the Gαi protein, rotigotine reduces the intracellular AC and cAMP activity, which in turn inhibits the phosphorylation function of PKA (Ford et al., 1996). Some studies have shown that the inflammatory response is closely related to the occurrence of PD (Machado et al., 2016). The inflammatory response is usually accompanied by the upregulation of COX-2 expression and the increased production of PGE2. COX-2 is an important inflammatory factor, which plays an important role in the pathogenesis of PD (Neves et al., 2015). Studies have shown that when peripheral nerves are damaged, the expression of COX-2 can be induced by the activated microglia and pro-inflammatory cytokines (Chu et al., 2015). COX-2 catalyzes the rate-limiting step in the production of prostaglandin (PG) by using arachidonic acid as the substrate (Torreilles et al., 1999). The production of PG leads to central sensitization and subsequently produces persistent pain (Samad et al., 2001; Wang and Wang, 2017).

In the experiments, the levels of cAMP, PKA, COX-2, and PGE2 in inflamed tissues were assayed after RTGT-MS administered. The results showed that a single intramuscular injection of 40 mg/kg RTGT-MS could reduce the carrageenan-induced increase of COX-2 and PGE2 levels and decrease the expression of cAMP and PKA in the inflamed tissues. Chlorpromazine attenuated the downregulation of COX-2 and cAMP induced by RTGT-MS, and it partially inhibited the downregulation of PKA. The co-administration of RTGT-MS and chlorpromazine still exerted a partial antinociceptive effect in the inflammatory pain model because chlorpromazine did not fully block RTGT-MS stimulation of the dopamine receptor. As shown in the results, it is reasonable to conclude that the antinociceptive effect of RTGT-MS on inflammatory pain is related to its stimulation of the dopamine receptors, at least, in part. However, the selective antagonists of dopamine receptors, which may block the central and peripheral dopaminergic receptors, need to be used to identify which dopamine receptor and how much to be associated with the antinociceptive effect of RTGT-MS.

Further, when the dopamine receptors were blocked by chlorpromazine, the reduction of COX-2, cAMP, PKA, and PGE2 levels induced by RTGT-MS was eliminated. Therefore, it can be inferred that the analgesic mechanism of RTGT-MS in the carrageenan-induced inflammatory pain model may be related to the AC-cAMP-PKA pathway and the peripheral D2 receptor.

Pain is associated with Parkinson’s disease itself and comorbid conditions. Rotigotine transdermal patch has been recommended by the International Parkinson and Movement Disorder Society Evidence-Based Medicine Committee in the treatment of non-motor fluctuations dominated by pain (Dutra et al., 2015; Seppi et al., 2019). In this study, our results show that RTGT-MS had an analgesic effect on carrageenan-induced inflammatory pain, suggesting that RTGT-MS may be used to relief the pain of musculoskeletal and joint caused by rigidity, akinesia or dystonia, and the pain of rheumatic inflammatory diseases in PD patients.

It should be point out that NSAIDs, as analgesic agents, are used not only to PD patients but also to comorbid conditions, such as, rheumatological diseases, osteoarthritis, etc. Celecoxib is a selective COX-2 inhibitor for treating rheumatic inflammatory diseases. In experiments, RTGT-MS and celecoxib were co-administered to determine whether these two drugs had a synergistic analgesic effect. The results showed both intramuscular injection of RTGT-MS in the analgesic dose range (3.9–40.7 mg/kg) and oral celecoxib in the range of 12–125.83 mg/kg could reduce pain induced by thermal and mechanical stimulation in a dose-dependent manner. Isobolographic analysis shows that the ED50 for the combination of these two drugs was significantly lower than the theoretical point fitted by the ED50 of the two drugs. These results indicate there is an interaction between these drugs, and the concurrent intramuscular administration of RTGT-MS and oral administration of celecoxib exerts a significant synergistic effect against carrageenan-inflammatory pain.

The findings provide the potential use of RTGT-MS to attenuate the pain, with advantage having continuous effect for more than 4 days at least each administration. RTGT-MS and celecoxib co-administered having a synergistic analgesic effect make it is possible to exert much more efficacy to relief the pain and reduce the dose of celecoxib. Note that this is a pre-clinical study on animal models, not clinical trials. Therefore, further studies involving humans should be performed.

## Conclusion

The findings suggest that RTGT-MS exerted analgesic effects on inflammatory pain, and the analgesic mechanism might be associated with regulating the AC-cAMP-PKA pathway through peripheral D2 receptor partly.

## Data Availability Statement

The original contributions presented in the study are included in the article/supplementary material, further inquiries can be directed to the corresponding author/s.

## Ethics Statement

The animal study was reviewed and approved by Laboratory Animal Ethics Committee of Yantai University with the authorization number of 20140901-01.

## Author Contributions

KL designed and conducted the study and drafted the original manuscript. FF revised the manuscript. FF and TW supervised the study. YZ, ET, and ZL contributed to the formal analysis and data curation. All authors approved the final manuscript.

## Funding

The study was supported by the Foundation of Collaborative Innovation Center of Advanced Drug Delivery System and Biotech Drugs in Universities of Shandong (Yantai University) and the Taishan Scholar Project of Shandong Province.

## Conflict of Interest

The authors declare that the research was conducted in the absence of any commercial or financial relationships that could be construed as a potential conflict of interest.
